# Superior mesenteric artery aneurysm endovascular repair

**DOI:** 10.1016/j.jvscit.2023.101227

**Published:** 2023-05-23

**Authors:** Antonio Solano, K. Benjamin Lee, Jesus Porras-Colon, Carlos H. Timaran, Vivek Prakash, Khalil Chamseddin, Melissa L. Kirkwood, M. Shadman Baig

**Affiliations:** Division of Vascular and Endovascular Surgery, Department of Surgery, University of Texas Southwestern Medical Center, Dallas, TX

**Keywords:** Aneurysm, Endovascular repair, Endovascular techniques, Superior mesenteric artery, Superior mesenteric artery aneurysm, Visceral artery aneurysm

## Abstract

Superior mesenteric artery aneurysms are rare and associated with high mortality rates in cases of rupture. Current Society for Vascular Surgery guidelines recommend treatment of all superior mesenteric artery aneurysms regardless of size. A 53-year-old woman who was admitted for abdominal pain was found with a 14-cm, ruptured superior mesenteric artery branch aneurysm. Endovascular approach was performed with microvascular plug embolization of a feeding branch and aneurysm sac exclusion with a stent graft. Four months later, the patient demonstrated a 21% regression of the aneurysm and stent patency. Thus, timely diagnosis and treatment of superior mesenteric artery aneurysms with endovascular techniques can reduce potential complications.

Superior mesenteric artery (SMA) aneurysms (SMAAs) are rare, representing 6% to 15% of all visceral artery aneurysms (VAAs).^1^ Unruptured aneurysms commonly present in asymptomatic patients, with incidental diagnosis through imaging.^2^^,^^3^ SMAA rupture and mortality rates are 38% to 50% and 30% to 90%, respectively.^2^^,^^4^ True aneurysms are defined by involvement of all three layers of the vessel wall and focal dilation 1.5 times greater than the size of the normal vessel, whereas pseudoaneurysms are contained ruptures lined by adventitia or perivascular tissues.^5^^,^^6^ The Society for Vascular Surgery guidelines recommend repair of both SMA pseudoaneurysm and true aneurysms as soon as the diagnosis is confirmed, regardless of size.^2^^,^^4^ The patient presented here provided written informed consent for the report of her case details and imaging studies.

## Case report

**Fig 1:**
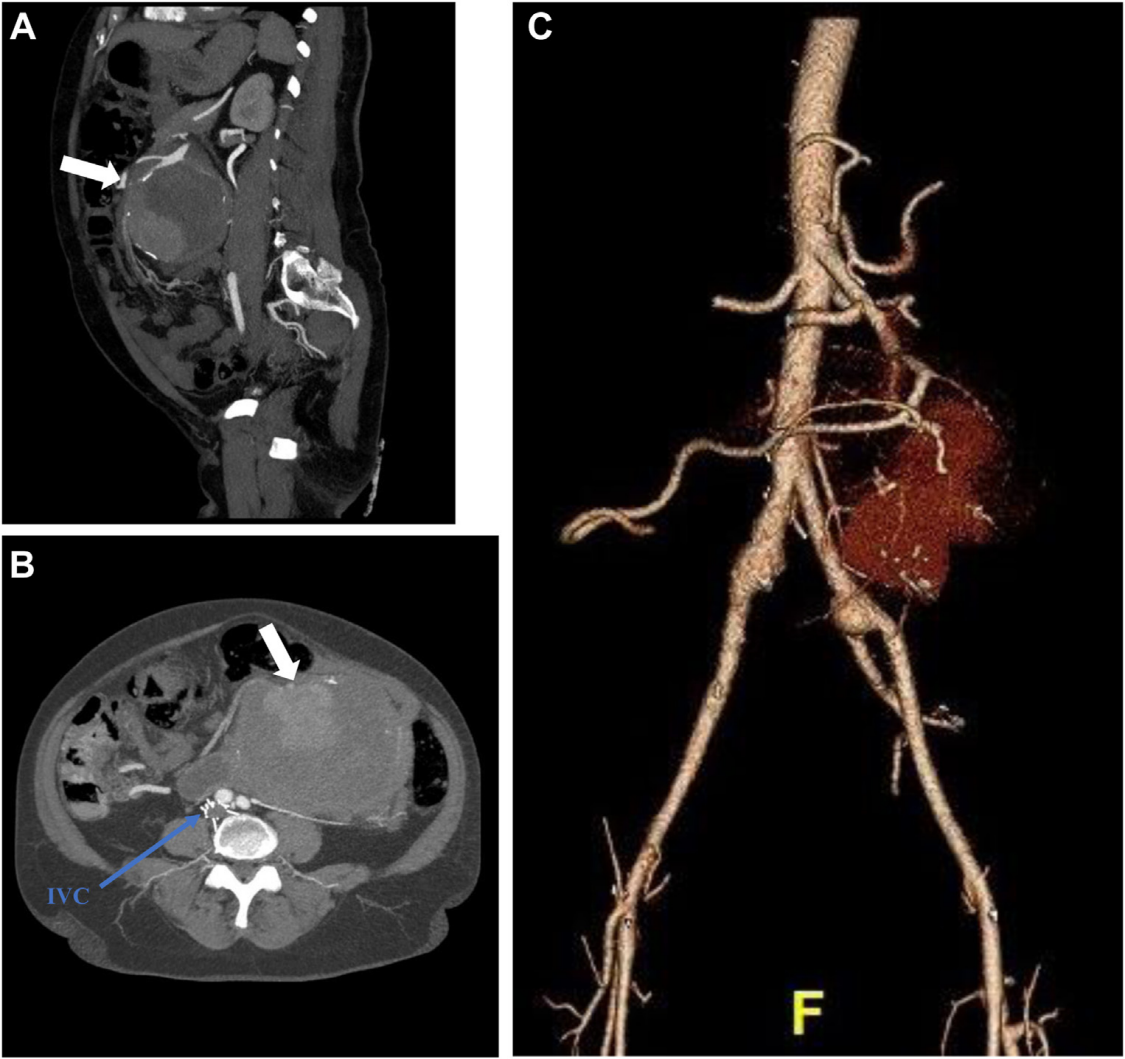
**A-C,** Sagital and axial view of abdominal computed tomography angiogram (CTA) demonstrating extensive surrounding mural thrombosis of a superior mesenteric artery (SMA) branch and feeding vessels (*arrow*). Three-dimensional preoperative reconstruction of SMA aneurysm. *IVC*, Inferior vena cava.

A 53-year-old female presented to an outpatient clinic with a history of several months of abdominal pain and distension. Past medical history was significant for hypertension, asthma, chronic anemia with iron supplementation, and bilateral below-knee amputations secondary to complicated meningitis in 2010. She also had a remote history of two mitral valve replacements, pacemaker placement, deep venous thrombosis, and inferior vena cava filter placement, and she was not on anticoagulation. The patient was evaluated by gastroenterology for mid-abdominal pain, foul smelling stools, and intermittent diarrhea. She underwent upper and lower endoscopy with no remarkable findings. Due to persistent symptoms over several months after negative endoscopy, a computed tomography (CT) scan was obtained, which revealed a 14 × 11 cm aneurysm arising from a jejunal branch of the SMA, with extensive surrounding mural thrombus (Fig 1). There was also an 18-mm aneurysm of bilateral common iliac arteries and a 6-mm saccular aneurysm of the left renal artery near the hilum. The patient was referred for urgent hospitalization and vascular surgery consultation. Upon presentation to the emergency department, she complained of chronic abdominal pain with fullness in the left upper quadrant. Her pain was exacerbated by eating, but there had been no acute change over the last few months. She was afebrile, white blood cell count was 6000/mL, hemoglobin was 9.1 g/dL with a mean corpuscular volume of 77 fL, and electrolytes showed mild hypercalcemia only. Lipase was slightly elevated (71 U/L), but serum amylase level was normal. There was low suspicion for a mycotic aneurysm due to the chronicity of her symptoms and lack of any systemic signs of infection or inflammatory changes in the surrounding mesentery. Our primary concern based on the imaging studies was a possible mesenteric mass having eroded into the vessel wall or an arteriopathy with a rupture of a saccular aneurysm. Due to the large nature of the aneurysm of a SMA branch and difficulty in visualizing the distal branches of the aneurysmal vessel on CT, we elected to proceed with angiography with possible embolization or stenting depending on angiographic findings.

**Fig 2:**
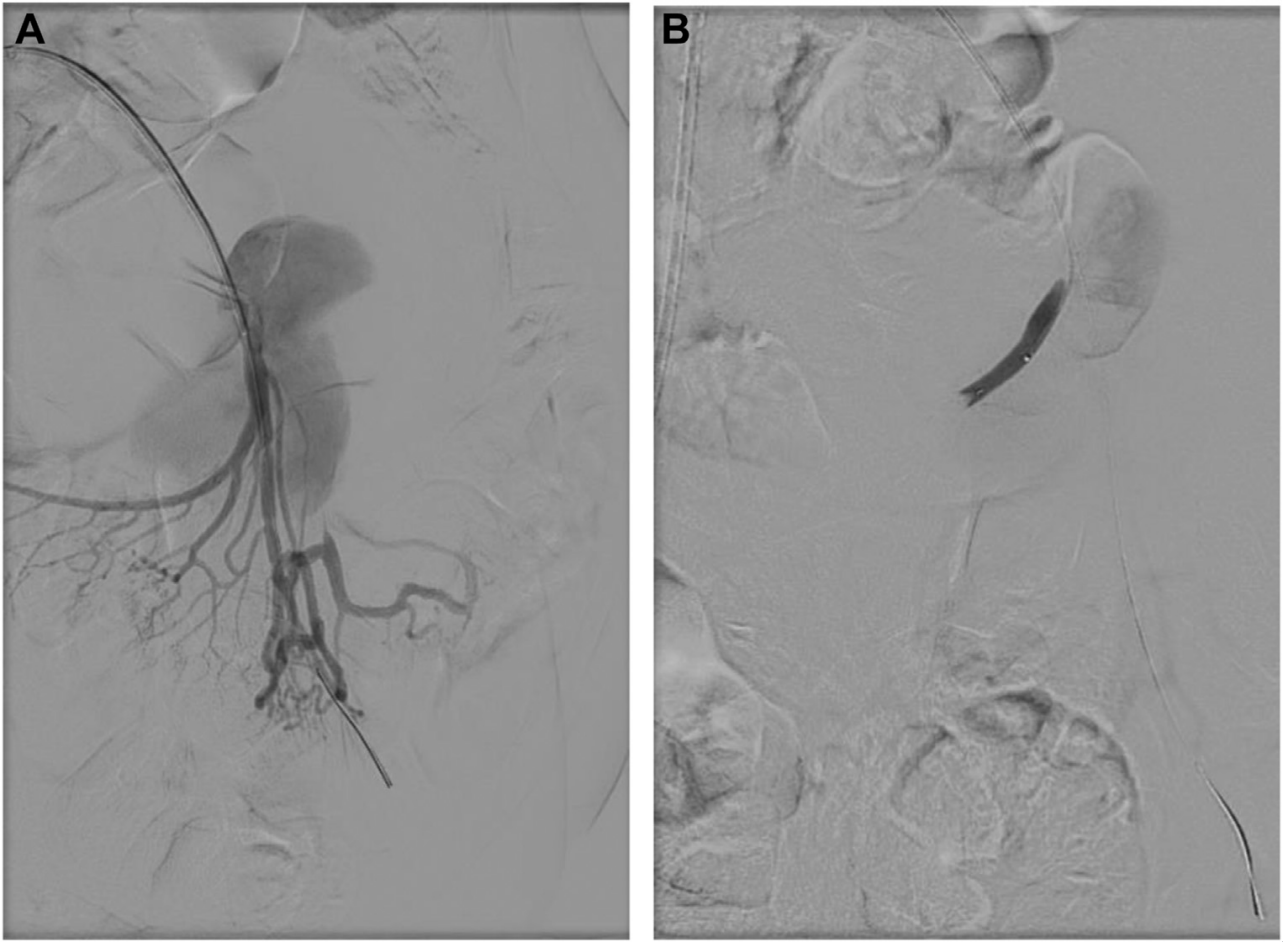
**A and B,** Operative angiogram sequence showing selective cannulation of the superior mesenteric artery (SMA). An aneurysmal mass with arterial extravasation off a branch from the SMA can be seen. One of the main branch vessels was embolized with a microvascular plug to avoid potential endoleak and facilitate the aneurysm sac exclusion.

**Fig 3:**
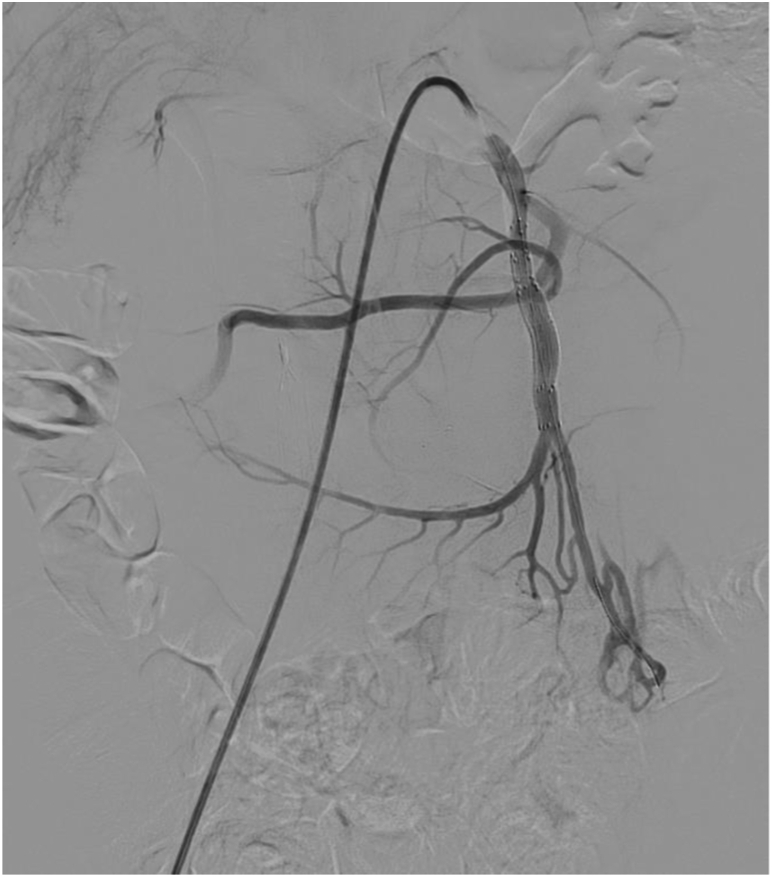
Completion angiogram with successful embolization of aneurysm sac.

**Fig 4:**
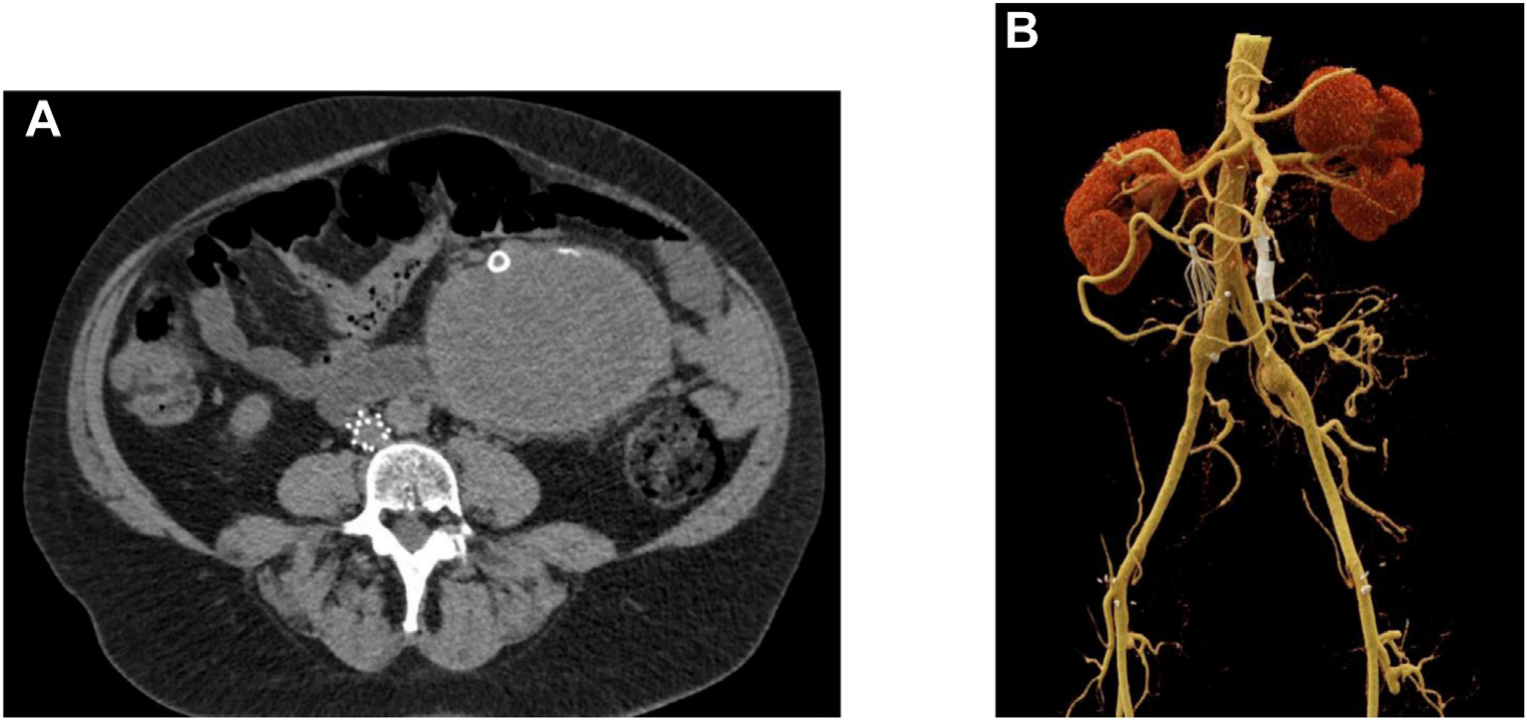
**A and B,** Axial view of follow-up abdominal computed tomography angiography (CTA) with aneurysm sac regression and superior mesenteric artery (SMA) stent patency. Three-dimensional postoperative reconstruction of SMA endovascular repair.

Under general anesthesia, percutaneous right femoral access was obtained with a 6F sheath. The SMA was cannulated using a coaxial system of a guiding catheter and a microcatheter through a steerable sheath. Angiography was performed to identify the aneurysm as well as the branch vessels. The common hepatic artery originated from the SMA. There was a large, likely pseudoaneurysm of a jejunal branch with displacement of the main trunk of the SMA. The branch vessel distal to the aneurysm was catheterized and angiography revealed a dilated distal branch that was perfusing a significant portion of the jejunum, making coil embolization prohibitively risky. A 0.014-in stiff guidewire was then placed distally. Next, a 0.014-in platform intravascular ultrasound catheter was advanced, and the vessel was interrogated. There was disruption of one wall of the jejunal branch at a branch point, indicating either a pseudoaneurysm or rupture of a saccular aneurysm at the branch point. Diameters of the vessel were obtained with intravascular ultrasound, and the distal jejunal artery (JA) was found to be dilated to 7.5 mm, whereas the more proximal JA measured 5 mm. The branch vessel off the JA was selectively catheterized with a buddy wire. Angiography of this branch of the JA demonstrated retrograde flow in the branch, indicating adequate collateralization from the main trunk of the SMA. It was embolized with a Microvascular Plug 5 (Medtronic) (Fig 2). Next, the 0.014-in wire in the main JA was exchanged for a 0.035-in TAD2 wire (Abbott Vascular) and a 6 mm × 2.5 cm Gore Viabahn self-expanding stent graft (WL Gore & Associates) was placed proximally in the JA, followed by an 8 mm × 5 cm Viabahn stent distally and a second 8 mm × 5 cm Viabahn stent to bridge between the two stents. Completion angiogram showed successful exclusion of the aneurysm with preservation of flow to the distal branches (Fig 3). Postoperatively, the patient had a decrease in her hemoglobin to 7 g/dL and received a transfusion. CT angiogram (CTA) was repeated and showed complete exclusion of the aneurysm with no bleeding complication. Over the next 2 days, she had significant improvement of the initial presenting abdominal pain and was tolerating a regular diet. She was discharged home on postoperative day 4 on aspirin 81 mg QD and clopidogrel 75 mg QD. Follow-up CTA at 4 months postoperatively demonstrated stent patency and aneurysm sac regression from 14 cm to 11 cm (Fig 4), with complete symptomatic relief. The patient will continue to have yearly monitoring with CT imaging.

## Discussion

DeBakey and Cooley performed the first SMAA resection in 1949 for a mycotic SMAA secondary to bacterial endocarditis.^7^ Despite infection being considered the most common etiology of SMAAs, current evidence has reported degenerative disease, connective tissue disease, and inflammatory conditions (pancreatitis, vasculitis, spontaneous dissection, fibromuscular dysplasia, and polyarteritis nodosa) as potential causes.^8^ Patients may present with abdominal and back pain and anemia, and diagnosis may be mistaken for other diseases such as pancreatitis, perforated viscus, ulcer disease, or appendicitis.^4^^,^^9^ In this case, initial clinical suspicion for a mycotic aneurysm was low. Gastrointestinal malignancy with erosion into the vasculature was considered and ruled out with further imaging of the abdomen with magnetic resonance imaging as well as laboratory studies of tumor markers carcinoembryonic antigen, CA 19-9, CA-125, and alpha-fetoprotein. Extensive infectious workup was deferred, given absence of inflammatory response, chronicity of clinical symptoms, and absence of fever or leukocytosis.

Per Society for Vascular Surgery guidelines for VAAs, all SMAAs and pseudoaneurysms should be repaired regardless of size.^2^ The exclusion of the aneurysmal sac from the systemic circulation while preserving distal blood flow is the goal of treatment. Despite consideration of open repair as the standard of care for many years, current trends have shifted to use of endovascular repair with covered stents and transcatheter embolization.^10^ Covered stents have demonstrated good results; however, when the aneurysm involves distal branches, there is a significant challenge in branch preservation, and open repair may be preferred over endovascular options in these cases.^2^^,^^11^ Embolization can be performed only in presence of sufficient collateral circulation and requires close patient postoperative monitoring.^6^ Considering a minimally invasive nature of endovascular therapy vs open surgery, it may provide advantages in difficult clinical scenarios such as abdominal sepsis or pancreatic inflammation.^12^

In this case, we elected to proceed with endovascular repair due to the large nature of the aneurysm involving the branches of the SMA and concern for safely obtaining control of all branches prior to entering the aneurysm cavity. Additionally, there was concern that the aneurysm could be secondary to a mesenteric mass eroding into the vasculature. Magnetic resonance imaging to rule this out was performed after repair. Evidence for VAA endovascular repair reports success rates between 93.3% and 98.3%, with major and minor complications in 1.8% to 3.6% and 8.9% to 10.5% of cases, respectively. Procedure-related complications include hematoma, vessel dissection, splenic infarction, intestinal ischemia, aneurysm reperfusion, and in-stent thrombosis.^10^^,^^13^ Sachdev et al^14^ reported the comparison of surgical and endovascular therapy for aneurysms involving branches of the celiac artery and SMA in 59 patients and demonstrated predominancy of an endovascular approach and shorter in-hospital stay vs open repair (2.4 vs 6.6 days; *P* < .001). Given these outcomes, endovascular treatment may be considered for select cases.

Average SMAA diameters range from 1.5 to 3 cm, but the literature has reported dimensions up to 4 to 8 cm.^15^ One group has reported a SMAA with dimensions similar to the one reported here, but it was treated with surgical repair.^16^ To date, this is the largest SMAA to be successfully treated via endovascular approach.

Monitoring of SMAAs and pseudoaneurysms with CTA is suggested 1 to 3 months after repair, followed by 1- or 2-year follow-up with CTA or Doppler ultrasound to evaluate for endoleaks and stent patency.^17^

## Conclusions

SMAA is a rare clinical entity. Prompt surgical repair prevents risk of rupture and mortality. Surgical approach should be individualized according to patient comorbidities and anatomy, with endovascular approach considered as a safe alternative. Follow-up with diagnostic imaging is required for aneurysm- or procedure-related complications.
